# Polymyxin B Inadequately Quenches the Effects of Contaminating Lipopolysaccharide on Murine Dendritic Cells

**DOI:** 10.1371/journal.pone.0037261

**Published:** 2012-05-18

**Authors:** Graham A. Tynan, Anne McNaughton, Andrew Jarnicki, Takao Tsuji, Ed C. Lavelle

**Affiliations:** 1 Adjuvant Research Group, School of Biochemistry and Immunology, Trinity Biomedical Sciences Institute, Trinity College, Dublin, Ireland; 2 Immunology Research Centre, Trinity Biomedical Sciences Institute, Trinity College, Dublin, Ireland; 3 School of Biomedical Sciences and Pharmacy, Faculty of Health, The University of Newcastle, Callaghan, Australia; 4 Department of Microbiology, School of Medicine, Fujita Health University, Aichi, Japan; National Jewish Health and University of Colorado School of Medicine, United States of America

## Abstract

Dendritic cell (DC) activation is commonly used as a measure of the immunomodulatory potential of candidate exogenous and endogenous molecules. Residual lipopolysaccharide (LPS) contamination is a recurring theme and the potency of LPS is not always fully appreciated. To address this, polymyxin B (PmB) is often used to neutralise contaminating LPS. However, the limited capacity of this antibiotic to successfully block these effects is neglected. Therefore, this study aimed to determine the minimum LPS concentration required to induce murine bone marrow-derived dendritic cell (BMDC) maturation and cytokine secretion and to assess the ability of PmB to inhibit these processes. LPS concentrations as low as 10 pg/ml and 20 pg/ml induced secretion of interleukin (IL)-6 and tumor necrosis factor (TNF)-α respectively, while a concentration of 50 pg/ml promoted secretion of IL-12p40. A much higher threshold exists for IL-12p70 as an LPS concentration of 500 pg/ml was required to induce secretion of this cytokine. The efficacy of PmB varied substantially for different cytokines but this antibiotic was particularly limited in its ability to inhibit LPS-induced secretion of IL-6 and TNF-α. Furthermore, an LPS concentration of 50 pg/ml was sufficient to promote DC expression of costimulatory molecules and PmB was limited in its capacity to reverse this process when LPS concentrations of greater than 20 ng/ml were used. There is a common perception that LPS is heat resistant. However, heat treatment attenuated the ability of low concentrations of LPS to induce secretion of IL-6 and IL-12p40 by BMDCs, thus suggesting that heat-inactivation of protein preparations is also an ineffective control for discounting potential LPS contamination. Finally, LPS concentrations of less than 10 pg/ml were incapable of promoting secretion of IL-6 independently but could synergise with heat-labile enterotoxin (LT) to promote IL-6, indicating that reducing contaminating endotoxin concentrations to low pg/ml concentrations is essential to avoid misleading conclusions regarding candidate immunomodulators.

## Introduction

Lipopolysaccharide (LPS) is a major component of the outer membrane of Gram-negative bacteria. This pyrogen is essential in maintaining the structural integrity of bacteria and repelling antimicrobial responses. LPS is a potent endotoxin comprised of a repetitive glycan polymer referred to as the O-antigen, which is connected to the core polysaccharide. The core domain is directly attached to Lipid A which is a phosphorylated glucosamine disaccharide containing multiple fatty acids which serve to secure the LPS molecule to the bacterial membrane. LPS is a pathogen-associated molecular pattern (PAMP) known to signal through the CD14/Toll-like receptor (TLR) 4/myeloid differentiated protein (MD)-2 receptor complex, thereby promoting innate immune responses characterised by rapid secretion of proinflammatory cytokines and chemokines [1]–[4]. Importantly, the Lipid A moiety is mainly responsible for these immunostimulatory effects [5]–[6].

In studies assessing the effects of candidate microbial and endogenous immunomodulators, LPS contamination of sample preparations has proven to be a hugely significant problem, often resulting in misleading and inaccurate conclusions. For example, one need look no further than the ambiguities associated with many of the more established endogenous ‘danger signals’ identified thus far in order to gauge the magnitude of the problem concerning residual LPS contamination [7].

In 1999, an immunomodulatory role for high-mobility group box protein 1 (HMGB1) was proposed; stimulation of monocyte cultures with HMGB1 induced secretion of a range of proinflammatory mediators including tumor necrosis factor (TNF)-α, interleukin (IL)-1β, IL-1α, IL-1 receptor antagonist (ra), IL-6, IL-8, macrophage inflammatory protein (MIP)-1α and MIP-1β. HMGB1 has since been shown to play important roles in both innate and adaptive immunity [8]–[12]. However, recent studies have contradicted many of the above findings, demonstrating that a highly purified form of recombinant HMGB1 fails to induce cytokine secretion from mouse or human macrophages [13]. Furthermore, the direct proinflammatory activity of HMGB1 reported previously and described above is likely to be a result of contamination with bacterial components as most of these studies were carried out using recombinant HMGB1 derived from *Escherichia coli* (*E. coli*) expression systems. Heat shock protein (Hsp) 60, Hsp70, Hsp90 and glycoprotein (gp) 96 have all been reported to stimulate dendritic cell (DC) [14]–[17] and macrophage activation [16], [18]–[20]. However, Wallin and colleagues noted that highly purified murine liver Hsp70 failed to stimulate cytokine secretion from murine DCs. In contrast, a Hsp70 preparation contaminated with low levels of LPS induced cytokine secretion at concentrations as low as 50ng/ml, and these effects were not inhibited by PmB and were also heat-sensitive [21]. In recent years many reports have been published which consistently outline the failure of highly purified Hsp molecules to stimulate immune responses in the absence of contaminating PAMPs. [21]–[24].

*In vitro*, heparan sulfate degradation products and hyaluronic acid fragments stimulate activation of DCs and endothelial cells [25]–[26]. However, many studies have reported a role for TLR4 [26]–[27], and to a lesser extent TLR2 [28], in regulating the alarmin function of these fragments. Despite these encouraging studies, caution must be warranted given that these fragments signal through TLR4. These concerns are heightened by the finding that highly purified pharmacological-grade hyaluronic acid fragments fail to induce NF-κB activation or cytokine secretion in murine macrophages [29].

DC activation, as measured by cytokine secretion and increased expression of costimulatory and major histocompatibility complex (MHC) molecules, is commonly used as a measure of the potency of candidate immunomodulators, particularly endogenous danger signals. However, it is important to note that many recombinant protein preparations are derived from bacterial expression systems. Thus, residual LPS contamination is a significant issue and the potency of LPS is not always fully appreciated. To address this, polymyxin B (PmB) is often used to neutralise contaminating LPS. However, the limited capacity of this antibiotic to successfully block these effects is neglected. Therefore, in order to characterise the immunomodulatory effects of candidate molecules with confidence, it is crucial to address this key issue. This study aimed to determine the minimum LPS concentration required to induce DC maturation and cytokine secretion, and to assess the ability of PmB to inhibit these processes.

## Materials and Methods

### Medium

Roswell Park Memorial Institute (RPMI) 1640 medium (Biosera) was supplemented with 2 mM L-Glutamine (Gibco), 50 units/ml penicillin (Gibco), 50 μg/ml streptomycin (Gibco) and 8% (v/v) heat-inactivated (56°C for 30 minutes) foetal calf serum (Biosera).

### Cell Culture

Female BALB/c mice were obtained from Harlan Olac (Bicester, U.K.) and were used at 8–16 weeks of age. Animals were maintained according to the regulations of the European Union and the Irish Department of Health. This study was approved by the Trinity College Dublin Animal Research Ethics Committee (Reference Number 091210). Bone marrow-derived dendritic cells (BMDCs) were generated as described previously [30]. Cells were plated out on day 10 and were stimulated on day 11. The specific treatments and conditions for stimulation of BMDCs are outlined in each experimental figure legend. Supernatants were collected 24 hours later for analysis of cytokine levels by enzyme-linked immunosorbent assay (ELISA). Following the removal of supernatants, the cells were harvested and assessed for toxicity and expression of surface markers by flow cytometry.

### Flow Cytometry

Cells were incubated for 30 minutes with AQUA fluorescent dye (0.5 μl) (Invitrogen) and then incubated for an additional 30 minutes with anti-CD11c (0.3 μg/ml, BD Pharmingen) and antibodies specific for CD80 (2.5 μg/ml), CD40 (2.5 μg/ml) and MHC class II (0.2 μg/ml) (BD Pharmingen). Samples were acquired using summit software (Dako, Colorado) and the data were analysed using FlowJo^TM^ software (Treestar, Oregon).

### Measurement of Cytokine Levels by ELISA

Concentrations of the cytokines IL-6, IL-12p40 and IL-12p70 were measured using ELISA antibodies obtained from BD Pharmingen. Concentrations of the cytokine TNF-α were measured using a commercially available ELISA kit (R&D systems). The OD values were obtained using a microtitre plate reader measuring absorbance at 492 nm, while the cytokine concentrations were determined using a standard curve.

### Polymyxin B

A range of concentrations of LPS from *E. coli*, Serotype R515, TLRgrade (Enzo Life Sciences) were incubated on a rotator for 2 hours at 37°C with 100 μg/ml PmB (Sigma). These treatments were then used to stimulate BMDCs.

### *Escherichia coli* heat-labile enterotoxin

*E. coli* heat-labile enterotoxin was kindly provided by Dr. Takao Tsuji (Department of Microbiology, School of Medicine, Fujita Health University, Aichi, Japan). This heat-labile enterotoxin was passed through three separate endotoxin removal columns (Pierce) three times each. Endotoxin level was subsequently measured using the limulus amebocyte lysate assay (Pierce) and was determined to be 0.049pg of endotoxin per μg of heat-labile enterotoxin.

### Statistical Analysis

Cytokine concentrations were compared by one-way ANOVA. Where significant differences were found, the Tukey-Kramer multiple comparisons test was used to identify differences between individual groups.

## Results

### Different thresholds exist for secretion of specific cytokines by DCs in response to LPS

**Figure 1:**
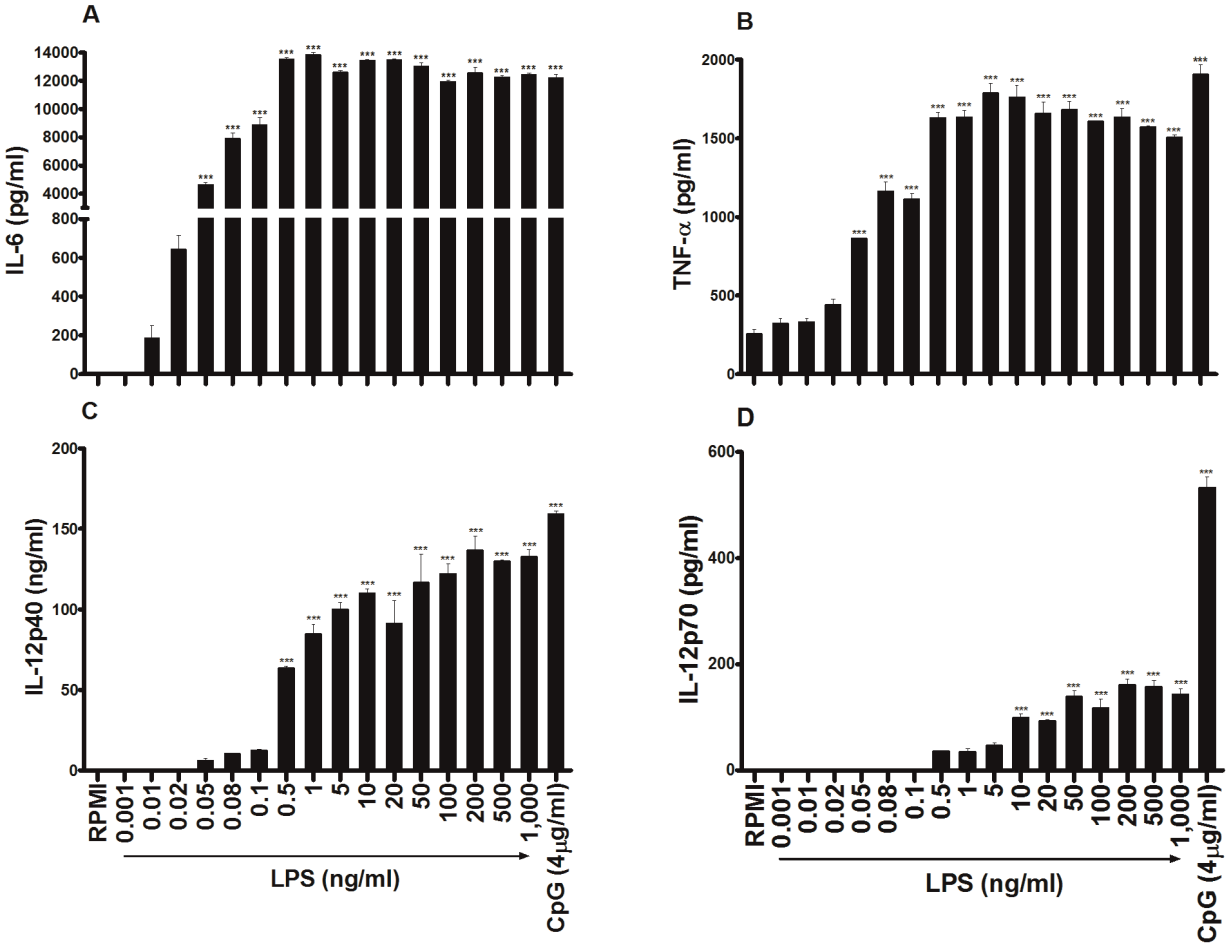
LPS concentrations as low as 10pg/ml induce secretion of proinflammatory cytokines by murine BMDCs. BMDCs (6.25×10^5^ cells/ml) from BALB/c mice were stimulated with concentrations from 1 pg/ml to 1µg/ml LPS. Supernatants were collected 24 hours later and tested for IL-6 (A), TNF-α (B), IL-12p40 (C) and IL-12p70 (D) by ELISA. Results are mean cytokine concentrations (+ SEM) for triplicate samples. Versus RPMI alone, *** p<0.001. Data are representative of three independent experiments.

In order to assess proinflammatory cytokine secretion by DCs in response to LPS, murine BMDCs were stimulated with concentrations from 1 pg/ml to 1 µg/ml *E. coli* LPS. LPS concentrations as low as 10 pg/ml and 20 pg/ml were sufficient to induce secretion of IL-6 (Figure 1A) and TNF-α (Figure 1B) respectively, while maximal secretion of these cytokines was evident when a concentration of 500 pg/ml LPS was added (Figure 1A, 1B).

LPS concentrations of 50pg/ml and 500 pg/ml were required to promote detectable secretion of IL-12p40 (Figure 1C) and IL-12p70 (Figure 1D) respectively, while maximal secretion of both of these cytokines was reached following stimulation with 50 ng/ml LPS (Figure 1C, 1D).

### A concentration of 50pg/ml LPS is sufficient to increase DC surface expression of MHC class II and costimulatory molecules

**Figure 2:**
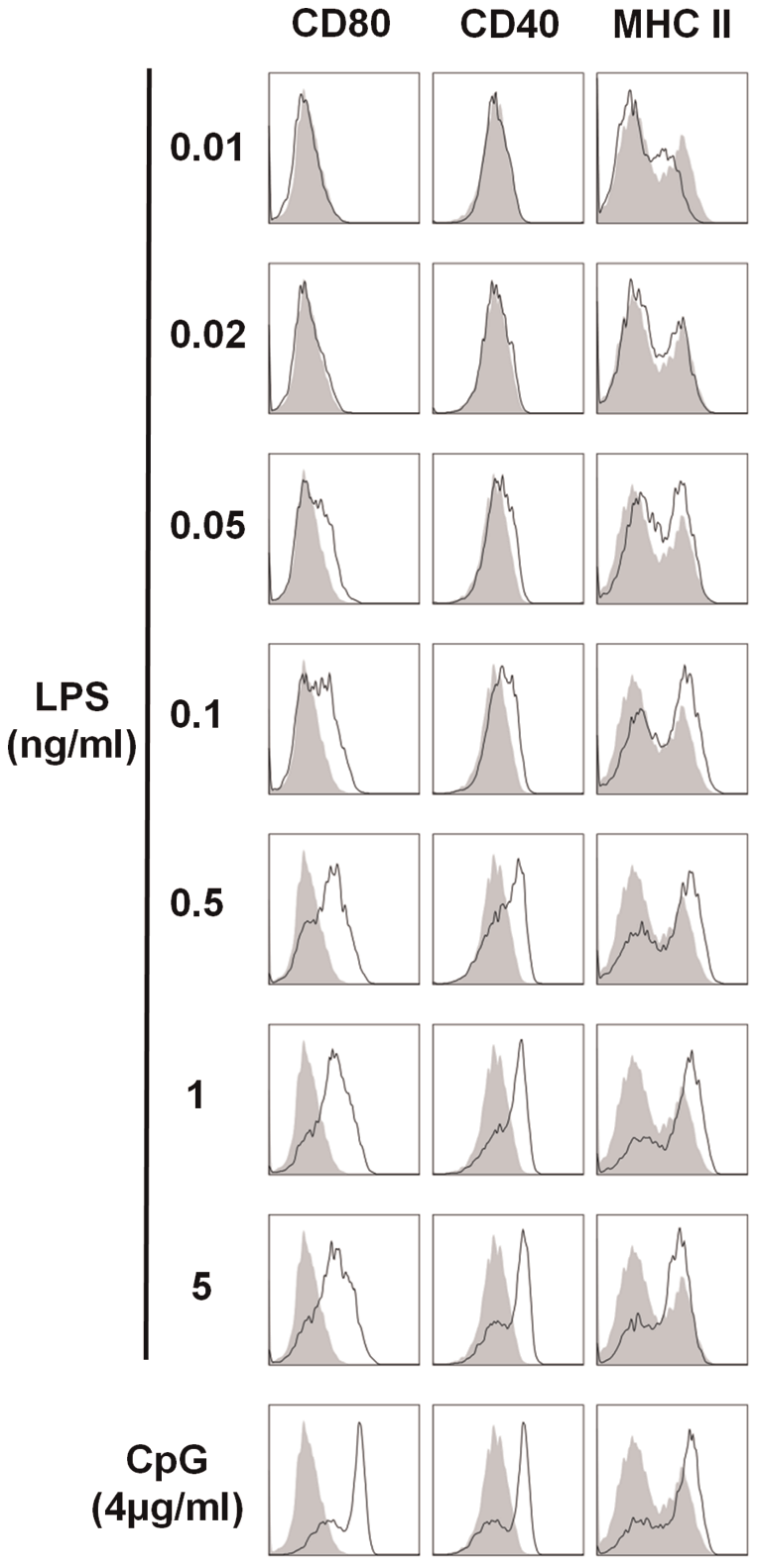
LPS concentrations as low as 50pg/ml increase expression of CD80, CD40 and MHC II on DCs. BMDCs were stimulated for 24 hours with the indicated concentrations of LPS and CpG. Cells were analysed for expression of CD80, CD40 and MHC II by flow cytometry. Immunofluorescence is shown for LPS- or CpG-treated BMDCs (black line) compared to untreated cells (grey histograms). Data are representative of three independent experiments.

DC activation is also characterised by increased expression of surface markers, including CD80, CD40 and MHC class II, in a process referred to as maturation. In order to evaluate the dose response of DC maturation by LPS, murine BMDCs were stimulated with concentrations from 1 pg/ml to 1 µg/ml LPS. A concentration as low as 50pg/ml LPS was sufficient to upregulate surface expression of CD80, CD40 and MHC class II (Figure 2). Furthermore, maximal DC maturation was achieved when an LPS concentration of 1ng/ml was used (Figure 2).

### 10μg/ml PmB is the optimal concentration for inhibiting LPS-induced DC activation

**Figure 3:**
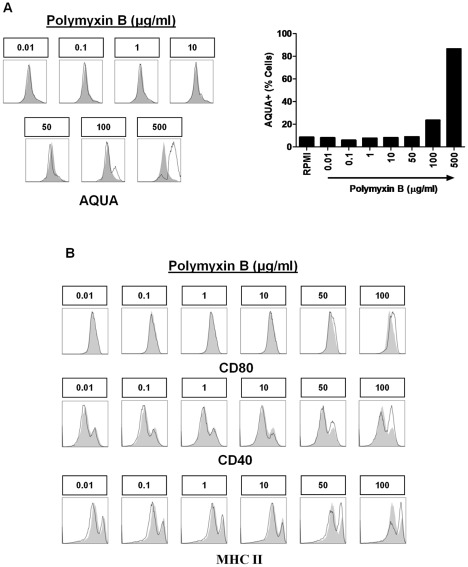
PmB concentrations greater than 10μg/ml are toxic and increase expression of costimulatory molecules on BMDCs. BMDCs were stimulated for 24 hours with the indicated concentrations of PmB. (A) Cells were stained with AQUA reactive dye and were analysed by flow cytometry. (B) Cells were also analysed for expression of CD80, CD40 and MHC II by flow cytometry. Immunofluorescence is shown for PmB-treated BMDCs (black line) compared to untreated cells (grey histograms). Data are representative of five independent experiments.

PmB is often used to neutralise contaminating LPS in sample preparations and therefore to eliminate the possibility that residual LPS may be responsible for any immunostimulatory effects observed. In order to determine the optimal PmB concentration for treating murine BMDCs without eliciting toxicity or maturation, these cells were stimulated with concentrations from 0.01 μg/ml to 500 μg/ml PmB. Notably, concentrations of 100 μg/ml PmB and higher were toxic to BMDCs (Figure 3A) and a concentration of 50 μg/ml PmB was sufficient to enhance expression of CD80, CD40 and MHC II (Figure 3B). Therefore, 10 μg/ml PmB was determined to be the optimal concentration for inhibiting LPS-induced BMDC activation without having any direct effect on the cells.

### The ability of PmB to reverse secretion of proinflammatory cytokines by DCs in response to LPS is cytokine-dependent

In order to assess the ability of PmB to inhibit proinflammatory cytokine secretion by DCs in response to LPS, murine BMDCs were stimulated with concentrations from 1pg/ml to 1µg/ml LPS, which were pre-incubated alone or in the presence of 100 μg/ml PmB for 2 hours at 37°C. Following pre-incubation of LPS with PmB, the final concentration of PmB added to the cells was 10 μg/ml.

**Figure 4:**
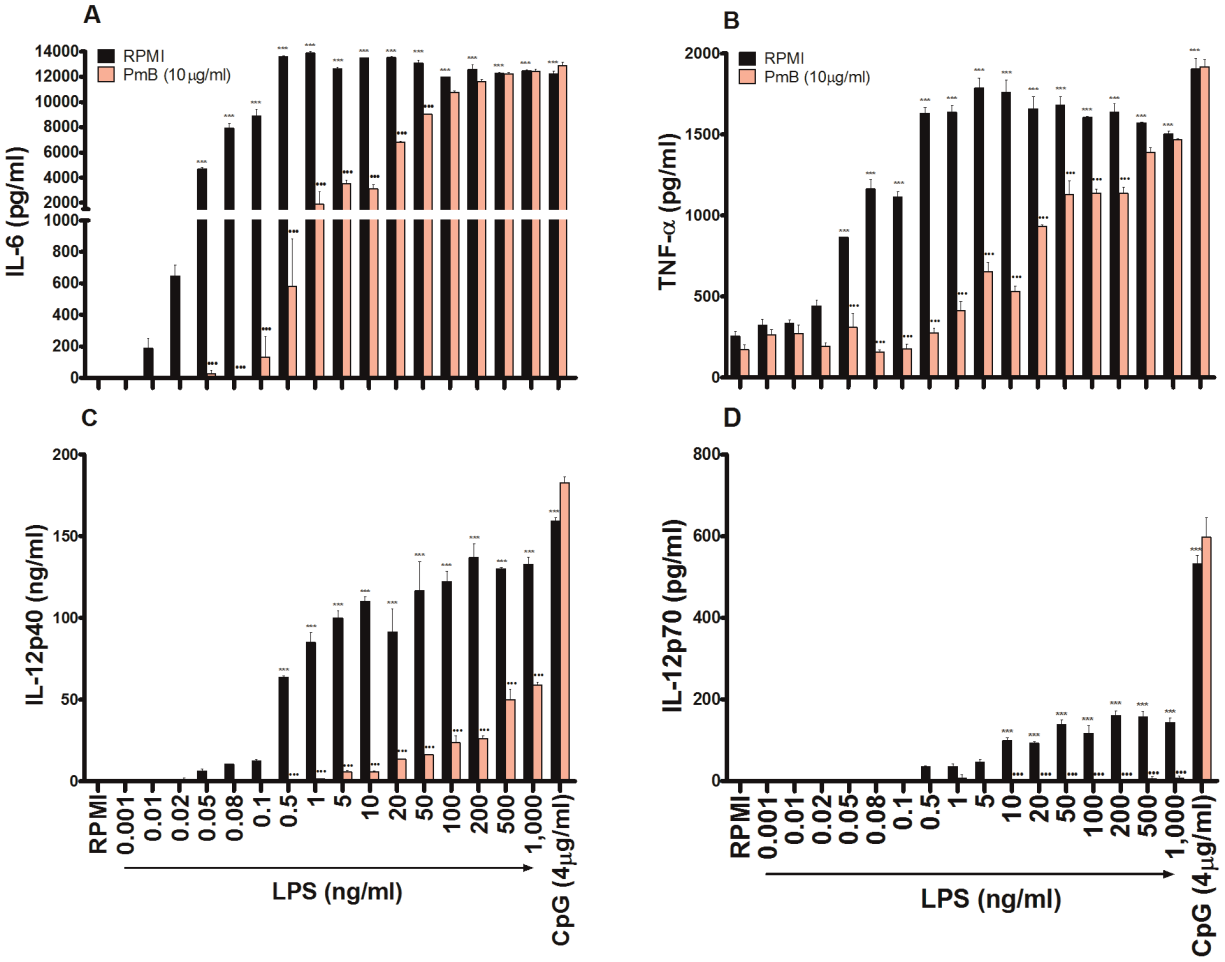
PmB exhibits a limited capacity to inhibit LPS-induced secretion of proinflammatory cytokines from BMDCs. BMDCs (6.25×10^5^ cells/ml) from BALB/c mice were stimulated with concentrations from 1 pg/ml to 1µg/ml LPS, which were pre-incubated alone or in the presence of PmB (100 µg/ml) for 2 hours at 37°C. Supernatants were collected 24 hours later and tested for IL-6 (A), TNF-α (B), IL-12p40 (C) and IL-12p70 (D) by ELISA. Results are mean cytokine concentrations (+ SEM) for triplicate samples. Versus RPMI alone, *** p<0.001, LPS + PmB versus LPS only, ••• p<0.001. Data are representative of three independent experiments.

The efficacy of PmB in suppressing secretion of proinflammatory cytokines was cytokine-dependent. When measuring IL-6, the inhibitory properties of PmB began to subside when LPS concentrations greater than 1 ng/ml were used. Furthermore, the ability of PmB to inhibit IL-6 secretion continued to decrease proportionally with increasing LPS concentrations until it was completely ineffective at concentrations greater than 50 ng/ml (Figure 4A). The capacity of PmB to inhibit LPS-induced TNF-α production was limited at LPS concentrations higher than 1 ng/ml. Moreover, following a steady decline in efficacy coinciding with increased concentrations of LPS, PmB was rendered entirely redundant when concentrations greater than 200 ng/ml LPS were used (Figure 4B). In contrast, the inhibitory properties of PmB on IL-12p40 and IL-12p70 were almost completely preserved until LPS concentrations of 20 ng/ml (Figure 4C) and 1µg/ml (Figure 4D) were used respectively.

### PmB is limited in its capacity to inhibit DC maturation in response to LPS

**Figure 5:**
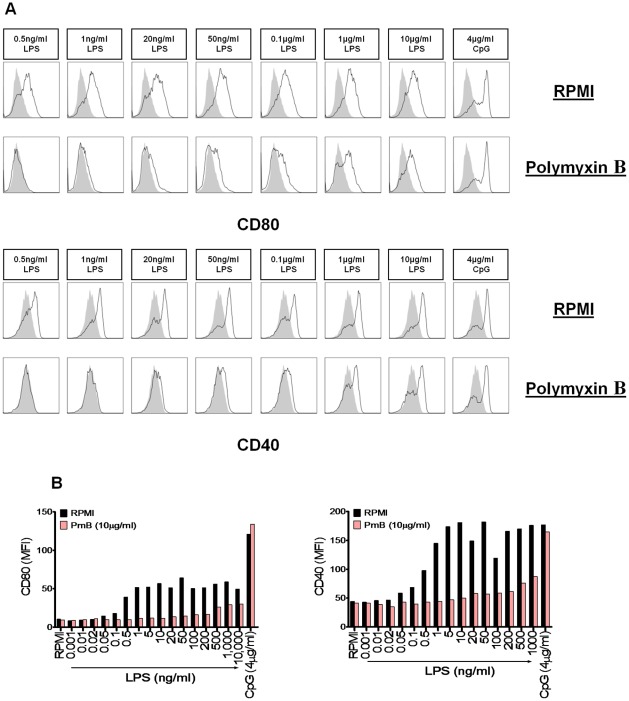
LPS-induced expression of CD80 and CD40 is no longer completely inhibited by PmB at a concentration of 20ng/ml. (A) BMDCs (6.25×10^5^ cells/ml) from BALB/c mice were stimulated with the indicated concentrations of LPS and CpG, which were pre-incubated alone or in the presence of PmB (100 µg/ml) for 2 hours at 37°C. Cells were harvested 24 hours later and analysed for expression of CD80 and CD40 by flow cytometry. Immunofluorescence is shown for LPS- or CpG-treated BMDCs (black line) compared to untreated cells incubated with medium (grey histograms). (B) BMDCs (6.25×10^5^ cells/ml) from BALB/c mice were stimulated as described in Figure 5A. Cells were harvested 24 hours later and analysed for expression of the maturation markers CD80 and CD40 by flow cytometry. Data is represented as median fluorescence intensity (MFI). Data are representative of three independent experiments.

In order to assess the ability of PmB to inhibit DC maturation in response to LPS, murine BMDCs were stimulated with concentrations from 1 pg/ml to 1 µg/ml LPS, which was pre-incubated alone or in the presence of 100 μg/ml PmB for 2 hours at 37°C. Again, the final concentration of PmB added to the cells was 10 μg/ml. Importantly, LPS-driven DC maturation was no longer completely inhibited by PmB at concentrations greater than 20 ng/ml (Figure 5A). Furthermore, the efficacy of PmB in suppressing this effect decreased proportionally with increasing concentrations of LPS (Figure 5B).

### Following heat treatment, low concentrations of LPS fail to promote secretion of the proinflammatory cytokines IL-6 and IL-12p40 by BMDCs

A second strategy used to determine if contaminating LPS is responsible for immunomodulatory effects is heat-inactivation of the protein [31]–[33]. Following heating to high temperatures a protein is presumed to become unstable and thus inactivated, whereas any LPS present in the preparation remains stable and intact. It can therefore be inferred that if the immunostimulatory response persists after heat-inactivation, it is a direct result of LPS contamination. Conversely, if the response is no longer observed after heat-inactivation then the protein is deemed to mediate the response rather than any contaminating LPS. However, Gao and colleagues have previously shown that following heat treatment, low concentrations of LPS fail to promote secretion of TNF-α by murine macrophages [22]–[23].

**Figure 6:**
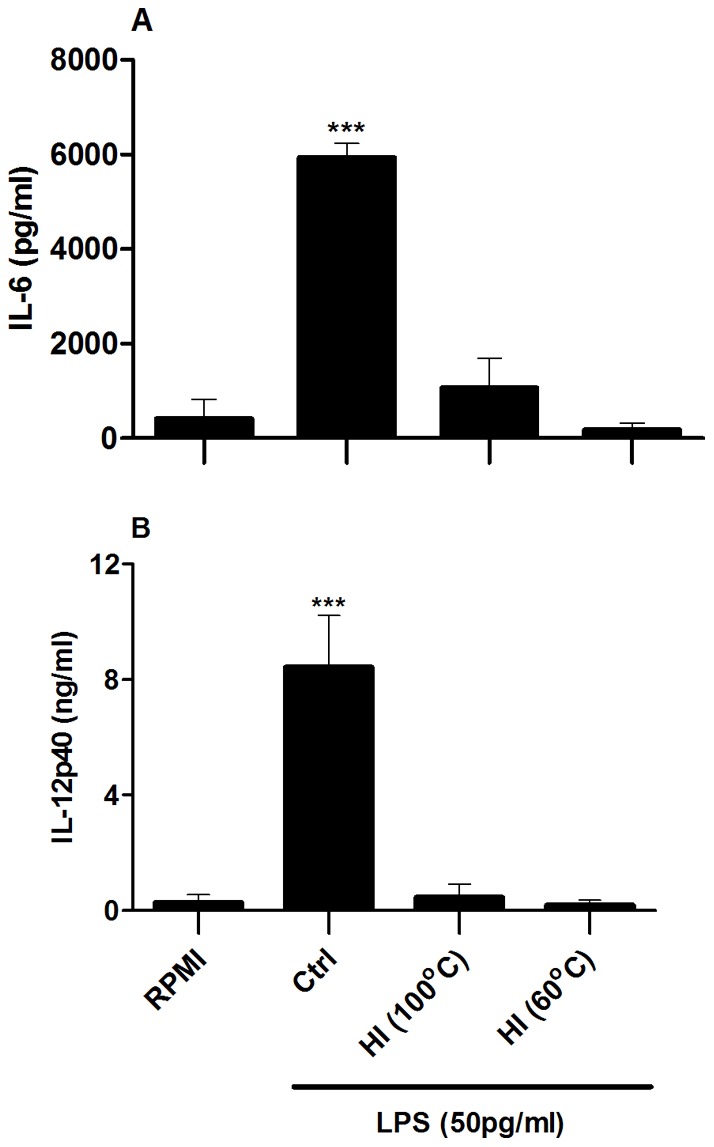
Heating low concentrations of LPS negates its ability to modulate BMDC secretion of IL-6 and IL-12p40. BMDCs (6.25×10^5^ cells/ml) from BALB/c mice were stimulated with control LPS (50 pg/ml) (Ctrl) or heat-inactivated (HI) LPS (100°C or 60°C for 1 hour). Supernatants were collected 24 hours later and tested for IL-6 and IL-12p40 by ELISA. Results are mean cytokine concentrations (+ SEM) for triplicate samples. Versus RPMI alone, *** p<0.001. Data are representative of four independent experiments.

As mentioned above, secretion of proinflammatory cytokines by BMDCs, particularly IL-6 and IL-12p40, is often used as a means to assess the immunomodulatory potential of candidate microbial and endogenous immunomodulators. In order to determine the effect of heat treatment on the ability of low concentrations of LPS to promote secretion of IL-6 and IL-12p40 by murine BMDCs, these primary cells were stimulated with LPS or heat-inactivated LPS (100°C or 60°C). A concentration of 50pg/ml LPS induced secretion of IL-6 and IL-12p40 by BMDCs, but this response was attenuated when heat-inactivated LPS was used (Figure 6).

### 5pg/ml LPS is sufficient to promote secretion of the proinflammatory cytokine IL-6 in the presence of *E. coli* heat-labile enterotoxin

Some of the more established endogenous danger signals, such as HMGB1, Hsps and small breakdown products of hyaluronic acid, have been reported to synergise with TLR agonists to promote a more potent inflammatory response than that generated against either component alone [34]–[37]. Furthermore, bacterial toxins including cholera toxin (CT), pneumolysin (PLY) and heat-labile enterotoxin (LT) can also elicit synergistic effects when used in combination with TLR agonists, in particular enhancing BMDC secretion of IL-6 [38]–[39]. This suggests that contaminating LPS concentrations which are incapable of promoting BMDC secretion of proinflammatory cytokines independently may synergise with candidate immunomodulators to enhance this effect, thus reducing even further the minimal LPS concentration required for BMDC activation.

**Figure 7:**
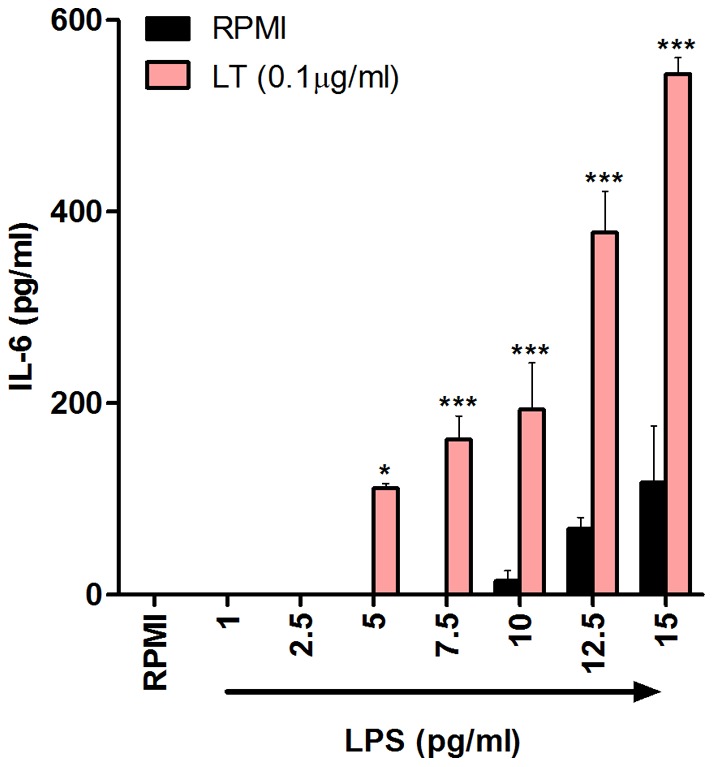
LPS concentrations as low as 5pg/ml induce secretion of IL-6 by murine BMDCs in the presence of *E. coli* heat-labile enterotoxin. BMDCs (6.25×10^5^ cells/ml) from BALB/c mice were stimulated with concentrations from 1pg/ml to 15pg/ml LPS, either alone or in the presence of LT (0.1 µg/ml). Supernatants were collected 24 hours later and tested for IL-6 by ELISA. Results are mean cytokine concentrations (+ SEM) for triplicate samples. LPS + LT versus LT only, * p<0.05 *** p<0.001. Data are representative of five independent experiments.

In order to establish if concentrations of LPS that are incapable of promoting cytokine secretion by BMDCs independently can synergise with a bacterial toxin to potentiate cytokine secretion, BMDCs were stimulated with suboptimal concentrations of LPS, either alone or in the presence of LT. Subsequently, BMDC secretion of IL-6 was measured. The presence of LT significantly augmented IL-6 secretion from BMDCs in response to as little as 5 pg/ml LPS which did not activate BMDCs in the absence of LT (Figure 7).

## Discussion

Dendritic cells are professional antigen-presenting cells (APCs) capable of linking innate and adaptive immunity. Following recognition of an activating stimulus, DCs initiate signal transduction cascades culminating in the synthesis and secretion of cytokines involved in promoting the onset of innate immunity and modulating subsequent effector T cell responses. Importantly, DCs have been classified into two discrete functional states, with each expressing a distinct phenotype. Immature DCs are highly efficient at antigen capture and processing, while mature DCs are potent APCs specialised for subsequent interactions with naive T cells [40]–[41]. DC activation is characterised by elevated surface expression of costimulatory molecules, including CD80 and CD40 as well as MHC molecules [42] in a process referred to as maturation. Interestingly, the term ‘mature’ is commonly used to describe DCs expressing high surface levels of costimulatory and MHC molecules, but is also used to describe immunogenic DCs capable of initiating adaptive immunity to foreign antigens. The discovery that non-immunogenic or immature DCs can also express elevated surface costimulatory and MHC molecules has been the main focus of recent reports which have questioned the apparent simplicity and transposable use of this terminology [42]. However, for the purpose of this study the term ‘maturation’ was used exclusively to define elevated expression of costimulatory and MHC molecules on the surface of BMDCs.

DC activation is often used as a means of determining the immunomodulatory properties of both PAMPs and host-derived danger signals. However, candidate danger signals are commonly purified from bacterial expression systems, particularly *E. coli*, and are frequently contaminated with bacterial PAMPs, especially LPS. Despite this problematic issue, many studies have failed to appreciate the potency of LPS and the profound ability of residual LPS contamination to induce DC activation.

In order to raise awareness of the potency of residual LPS, this study evaluated the dose response of DC activation by this PAMP. LPS concentrations as low as 10 pg/ml and 20 pg/ml were sufficient to induce BMDC secretion of the proinflammatory cytokines IL-6 and TNF-α respectively. Furthermore, a concentration of 50 pg/ml LPS was adequate to enhance expression of costimulatory and MHC II molecules. The magnitude of both costimulatory molecule expression and cytokine secretion increased dramatically with elevated concentrations of LPS. However, with regard to LPS-induced secretion of IL-6 and TNF-α, maximal activation was achieved by relatively low concentrations of LPS, more specifically concentrations upwards of 500 pg/ml. Therefore, when using these readouts to assess potential immune activation properties of candidate molecules, particularly endogenous danger signals, it is essential to almost completely eliminate all of the LPS in the preparation in order to circumvent artificial and misleading conclusions.

Interestingly, this study also found that secretion of IL-12p40 and particularly IL-12p70 by DCs is less sensitive to LPS than secretion of IL-6 and TNF-α or surface expression of costimulatory and MHC molecules. More specifically, LPS concentrations of 50 pg/ml and 500 pg/ml were required to promote minimal secretion of IL-12p40 and IL-12p70 respectively, while maximal secretion of these cytokines was achieved by stimulating DCs with 50ng/ml LPS. Therefore, there are different thresholds for secretion of specific cytokines by DCs in response to LPS. Ultimately, it is important for researchers to be vigilant of this when assessing the immune activation properties of candidate molecules.

The molecular weight of LPS can vary a great deal due to the heterogeneity within the acyl chains and differential phosphorylation. In order to address this problem, the U.S. Food and Drug Administration (FDA) have developed endotoxin units (EU) as an alternative to units of weight. EU describes the biological activity of LPS and a single EU is equal to approximately 100pg of *E. coli* LPS, the amount present in 1 x 10^5^ bacteria. Many research laboratories use EU values to determine contaminating LPS concentrations in sample preparations, particularly when using limulus amebocyte lysate testing. It is important to note that 10 pg/ml LPS is equivalent to as little as 0.1 EU/ml.

The literature contains many studies reporting novel immunostimulatory properties for endogenous molecules, many of which signal through TLR4. However, many of these reports fail to use effective controls for residual LPS contamination.

Perhaps the most common control used to eliminate LPS contamination is the *Bacillus polymyxa*-derived antibiotic PmB. PmB possesses a very high binding affinity for the lipid A moiety of LPS, thus serving to neutralise this PAMP and prevent interaction with TLR4 [43]. However, the limitations of PmB in reversing the effects of DC activation are often neglected. This study has shown that the assumption that the presence of PmB eliminates LPS-mediated effects on DCs is inaccurate, and its effects are dependent on both the concentration of LPS present and the particular cytokines under investigation.

Many studies reporting immunomodulatory activity for endogenous danger signals routinely use a concentration of 10 µg/ml PmB to antagonise residual LPS contamination [27], [44]–[48]. Indeed, higher concentrations of PmB are not normally used to inhibit LPS-mediated DC activation due to concerns over toxicity. By performing a dose response determination, this study has shown that 10 μg/ml PmB is an appropriate concentration for inhibiting LPS-induced DC activation without eliciting any adverse effects on the cells. 50 μg/ml PmB induced partial upregulation of CD80, CD40 and MHC II, whereas 100 μg/ml PmB was toxic. Importantly, in this study, LPS was pre-incubated with 100 µg/ml PmB for 2 hours at 37°C, thereby exposing LPS to PmB concentrations ten-fold greater than the 10 μg/ml optimal concentration determined here and also used in other studies. However, once added to the DCs, the final concentration of PmB was 10µg/ml which is non-toxic and does not enhance expression of costimulatory or MHC II molecules.

The efficacy of PmB varied for different cytokines and was ineffective for LPS concentrations greater than 20ng/ml in the case of IL-6 and TNF-α. Therefore, not only can extremely low concentrations of LPS induce secretion of IL-6 and TNF-α, but PmB is also exceedingly limited in its capacity to successfully reverse these effects. However, for IL-12p40 and IL-12p70, the inhibitory properties of PmB were preserved until LPS concentrations of 0.5 µg/ml and 1µg/ml were reached respectively. Despite this, LPS-driven DC maturation was no longer completely inhibited by PmB at LPS concentrations greater than 20 ng/ml, thereby further emphasising the inadequacies of this control.

The common perception that LPS is heat resistant dates back to the 19^th^ century when it was demonstrated by Richard Pfeifer that heat-inactivated *Vibrio cholera* retained their ability to induce shock in experimental animals. This led to a widely used method of boiling protein preparations for at least 30 minutes to rule out LPS contamination as the cause of the observed effects [33]. However, this study has shown that secretion of IL-6 and IL-12p40 by BMDCs in response to low concentrations of LPS is reduced by heating at 60°C or 100°C.

These results support other studies which have shown that low concentrations of heat treated *E. coli* LPS fail to promote TNF-α secretion by murine macrophages [22]–[23]. Importantly, Gao and colleagues outline that many studies use very high concentrations of LPS, approximately 10–500 ng/ml, to test for heat sensitivity. Since only minute concentrations of LPS are required to fully activate an APC, Gao *et* *al*. propose that even if practically all of the LPS is inactivated by heat treatment, the active residual LPS is sufficient to activate an APC, thus leading to the incorrect conclusion that LPS is heat resistant. In support of this idea, this study found that a concentration of 50 pg/ml LPS was sufficient to induce DC maturation and robust secretion of a number of proinflammatory cytokines. Therefore, LPS is capable of promoting DC activation at concentrations approximately two hundred times less than the minimal concentrations being used to test for heat sensitivity.

Many studies have reported that TLR agonists can synergise with microbial and endogenous immunomodulators to enhance BMDC secretion of specific proinflammatory cytokines, in particular IL-6 [34]–[39]. This study hypothesised that extremely low concentrations of LPS that are incapable of promoting cytokine secretion independently can synergise with candidate molecules to promote this effect. This ultimately generates the incorrect conclusion that the candidate molecule can induce cytokine secretion independently of LPS. Interestingly, this study shows that a concentration as low as 5 pg/ml LPS alone is not sufficient to promote secretion of IL-6 but can still synergise with LT to promote IL-6 secretion. Crucially, LT alone was unable to induce IL-6 secretion, thus raising the possibility that many proposed exogenous and endogenous immunomodulators elicit activity only as a direct result of synergy with low levels of contaminating LPS. Furthermore, the effects of contaminating LPS are also likely to result in misleading conclusions when using other APCs, including macrophages.

In conclusion, this study has revealed that extremely low concentrations of LPS can promote DC activation and that different thresholds exist for secretion of specific cytokines in response to this potent bacterial PAMP. Furthermore, PmB is limited in its capacity to reverse the effects of residual LPS contamination and is therefore an inadequate and unreliable control. In light of both the potency of LPS and the inability of PmB to efficiently neutralise this PAMP, it is necessary to completely remove LPS from candidate immunostimulatory molecules before using murine BMDC activation to test for potential activity. However, the complete removal of LPS from preparations is exceedingly difficult to accomplish, particularly for proteins such as HMGB1 and certain Hsps that bind specifically to this PAMP [35], [49]–[50]. Therefore, in cases where removal of LPS is not feasible we propose that the only definitive way to eliminate the possibility of residual LPS contamination is to use TLR4-deficient DCs or immortalised cell lines which do not express TLR4. However, the obvious drawback to this method is that genuine TLR4 agonists may evade discovery.

It is imperative that the potency of LPS in promoting DC activation is fully appreciated and the limitations of many of the controls for residual LPS contamination are not neglected. In order to reliably identify novel immunostimulatory molecules using dendritic cell models, these key issues must be addressed appropriately.
